# Thy‐1 knockdown promotes the osteogenic differentiation of GMSCs via the Wnt/β‐catenin pathway

**DOI:** 10.1111/jcmm.17955

**Published:** 2023-10-02

**Authors:** Gufeng Liu, Guixin Zhu, Xiaoyi Wu, Ziqiao Tang, Wenjun Shao, Min Wang, Haibin Xia, Quan Sun, Mingdong Yan

**Affiliations:** ^1^ State Key Laboratory of Oral & Maxillofacial Reconstruction and Regeneration, Key Laboratory of Oral Biomedicine Ministry of Education, Hubei Key Laboratory of Stomatology, School & Hospital of Stomatology Wuhan University Wuhan People's Republic of China; ^2^ The State Key Laboratory of Oral Diseases and National Clinical Research Center for Oral Diseases, Department of Prosthodontics, West China Hospital of Stomatology Sichuan University Chengdu China; ^3^ Department of Oral Implantology, Hospital and School of Stomatology Wuhan University Wuhan People's Republic of China; ^4^ Center for Prosthodontics and Implant Dentistry, Optics Valley Branch, School and Hospital of Stomatology Wuhan University Wuhan People's Republic of China; ^5^ Fujian Key Laboratory of Oral Diseases & Fujian Provincial Engineering Research Center of Oral Biomaterial & Stomatological Key lab of Fujian College and University, School and Hospital of Stomatology Fujian Medical University Fuzhou People's Republic of China

**Keywords:** mesenchymal stem cells, osteogenic differentiation, thy‐1, Wnt signalling pathway

## Abstract

Gingival mesenchymal stem cells (GMSCs) are newly developed seed cells for tissue engineering owing to their easy isolation, abundance and high growth rates. Thy‐1 is an important regulatory molecule in the differentiation of mesenchymal stem cells (MSCs). In this study, we investigated the function of Thy‐1 in the osteogenic differentiation of GMSCs by reducing the expression of Thy‐1 using a lentivirus. The results demonstrated that Thy‐1 knockdown promoted the osteogenic differentiation of GMSCs in vitro. Validation by RNA‐seq revealed an obvious decrease in *Vcam1* and *Sox9* gene expression with Thy‐1 knockdown. Kyoto Encyclopedia of Genes and Genomes pathway analysis suggested that the differentially expressed genes were enriched in the Wnt signalling pathway. We further demonstrated that Thy‐1 knockdown promoted osteogenic differentiation of GMSCs by activating the Wnt/β‐catenin signalling pathway. Therefore, Thy‐1 has a key regulatory role in the differentiation of GMSCs and maybe a core molecule connecting transcription factors related to the differentiation of MSCs. Our study also highlighted the potential of Thy‐1 to modify MSCs, which may help improve their use in tissue engineering.

## 1 INTRODUCTION

Mesenchymal stem cells (MSCs) are multipotent cells capable of differentiating into multiple mesenchymal lineages, including osteoblasts, adipocytes and chondroblasts.^1^, ^2^ Therefore, MSCs have recently attracted growing interest as promising cell types in the field of regenerative medicine.^3^ MSCs are present in the stroma of almost all organs. Among these, gingival mesenchymal stem cells (GMSCs) are one of the most conspicuous cells because of their easy isolation, abundance, high growth rate, strong self‐renewal and multi‐lineage differentiation ability.^4^, ^5^, ^6^ Some studies suggested that GMSCs might be superior to bone mesenchymal stem cells (BMSCs) for stem cell‐based regeneration therapy.^6^, ^7^ In our previous studies, GMSCs demonstrated higher proliferative and osteogenic abilities than BMSCs in vitro.^7^ The osteogenic effects of GMSCs are comparable to or even better than those of BMSCs in vivo.^6^ Therefore, in this study, we further investigated regulatory factors for the osteogenic differentiation of GMSCs.

Osteogenic differentiation of MSCs is regulated by multiple mechanisms involving several intracellular and intercellular signal transmission factors,^8^ such as growth factors, transcription factors, signalling pathways and microRNAs. The signalling pathways involved in the regulation of osteogenic differentiation include the Wnt/β‐catenin,^9^ transforming growth factor‐beta (TGF‐β)/bone morphogenetic protein (BMP),^10^ mitogen‐activated protein kinase (MAPK)^11^ and fibroblast growth factor (FGF) signalling pathways.^12^ Among these, the Wnt/β‐catenin signalling pathway plays a critical role in the proliferation, differentiation and bone formation of osteoblasts.^13^ Chen et al. used Dickkopf Wnt signalling pathway inhibitor 1 (DKK1) to suppress the Wnt/β‐catenin signalling pathway, which resulted in reduced proliferation, decreased osteoblastogenesis and matrix mineralization.^14^ With the Wnt/β‐catenin signalling pathway activation, released β‐catenin accumulates in the cytoplasm and eventually shifts into the nucleus, causing the activation of target genes.^15^ Conditional knockout of β‐catenin led to embryonic bone formation disorder because of osteoblast differentiation stasis at the early progenitor stage, with only alkaline phosphatase (ALP) and type I collagen expressed.^16^

Thy‐1, also known as the cluster of differentiation 90 (CD90), is a small glycoprotein composed of 110 amino acids anchored to the outer plasma membrane via glycophosphatidylinositol.^17^, ^18^ It is extensively distributed in fibroblasts, neurons, endothelial cells and thymocytes and has been considered a marker for identifying MSCs.^19^, ^20^ Some studies have suggested that Thy‐1 may not only be a marker for MSCs but also a key regulator of differentiation in endoderm, mesoderm and ectoderm‐derived cells.^21^, ^22^, ^23^ As a cell surface protein, Thy‐1 not only acts in the classical receptor/ligand interaction (*trans*)^17^ but also interacts with many molecules within the membrane of the same cell (*cis*).^23^ At present, the specific function of Thy‐1 remains unclear and seems to vary with cell type.^20^, ^24^ Accumulating evidence suggests that Thy‐1 is vital for the fate decision of MSCs. Picke et al. showed that osteoblast differentiation of BMSCs from Thy‐1‐deficient mice was lower than that of BMSCs from wild‐type mice.^25^ However, Moraes et al. reduced the expression of Thy‐1 in MSCs using Thy‐1‐target small hairpin RNA (shRNA) lentiviral vectors and found that the osteogenic differentiation of these MSCs was enhanced.^26^ Our previous study suggested that Thy‐1 may account for the differences in proliferative capacity and osteogenic potential between BMSCs and GMSCs.^7^

Given that Thy‐1 might be the reason for the special differentiation ability of GMSCs^7^ and that the specific function of Thy‐1 in MSC remains unclear,^26^ we knocked down the expression of Thy‐1 in GMSCs to detect its effect on the biomineralization of GMSCs in vitro. We used RNA sequencing (RNA‐seq) to determine the potential mechanism of Thy‐1 in the osteogenic differentiation of GMSCs. Finally, we studied the signalling pathways through which Thy‐1 regulates osteogenesis in GMSCs. The results of this study provide a comprehensive understanding of the functional effects of Thy‐1 on osteogenic differentiation and elucidate the regulatory mechanisms of osteogenic differentiation, providing novel insights into the optimization of GMSC osteogenic differentiation induction and promoting the development of stem cell‐based regenerative therapies and bone tissue engineering.

## 2 MATERIALS AND METHODS

### 2.1 Isolation of cells and cell culture

Gingival tissue was collected from the alveolar bone of 8‐week‐old C57BL/6 male mice brought from the Hubei Research Center of Laboratory Animals (Wuhan, China). The Ethics Committee for Animal Research, School and Hospital of Stomatology, Wuhan University approved all animal experimental procedures in the present study (protocol No. S0792203006). Gingivae were placed in phosphate‐buffered saline (PBS; Cytiva) with 100 U/mL penicillin/streptomycin/amphotericin B sterile solution (PSA; Solarbio). The gingivae were then cut into 1–2 mm^2^ small tissue blocks and digested with 0.1% Type I collagenase (BioFroxx) in Alpha Modified Eagle's Medium (α‐MEM, Cytiva) in a shaker at 180 rpm and 37°C for 1 h. After 1 h of enzymatic digestion, the suspension was centrifuged at 1000 rpm for 5 min to pellet the cells and tissues. Cell pellets were resuspended and inoculated in a T25 culture flask with α‐MEM (Minimum Essential Medium) containing 10% fetal bovine serum (FBS, Gibco) and 100 U/mL PSA. The cells were cultured in an incubator at 37°C and 5% CO_2_.

### 2.2 Flow cytometry

The second passage of GMSCs was used for flow cytometry analysis. Cells were harvested using trypsin‐ethylenediaminetetraacetic acid (Hyclone). After adding medium to quench the enzyme, the samples were centrifuged at 1000 rpm for 5 min. The cells were then resuspended in PBS with 1% FBS, and the concentration of cells was adjusted to 1 × 10^7^ cells/mL. The samples were incubated with the rat antibodies for mouse CD90 and CD105 conjugated fluorescein isothiocyanate (FITC) and mouse CD45 and CD19 conjugated phycoerythrin (PE) (eBioscience) in dark at 4°C for 30 min. Flow cytometric analysis was conducted using a FACSAria II flow cytometer (BD Biosciences).

### 2.3 Lentiviral transduction for Thy‐1 knockdown

To establish stable Thy‐1 knockdown cell lines, the second passage of GMSCs was placed in a 24‐well plate (3 × 10^5^ cells/well) with α‐MEM containing 10% FBS and 100 U/mL PSA. On reaching 40% confluence, the cells were transduced with non‐targeting shRNA‐expressing lentivirus or Thy‐1 shRNA‐expressing lentivirus at 50 multiplicity of infection (MOI) following the manufacturer's instructions (Hanbio). After 24 h of transduction, the culture medium containing lentivirus was replaced with a fresh medium. After 72 h, the medium with 4 μg/mL puromycin (BioFroxx) was used to select stable cell lines. After antibiotic selection for 3 weeks, stable Thy‐1‐knockdown cells (sh‐Thy‐1 group) and non‐targeting transduced cells (sh‐NC group) were obtained. These cells were observed under a fluorescence microscope (IX83, Olympus) to confirm that the shRNA sequence was successfully transfected into the cells based on their green fluorescent protein (GFP) fluorescence signals. The percentage of positive GMSCs was measured by automated cell counting function in ImageJ version 1.54d (National Institutes of Health, USA). After harvesting the cells, real‐time polymerase chain reaction (RT‐PCR) and western blotting were performed to measure the expression level of Thy‐1.

### 2.4 Mineralization induction

To exclude the effects of proliferation, GMSCs from each group were seeded at a high density (1 × 10^5^ cells/cm^2^). After the cell density reached 80%, the growth medium was replaced with the mineralization‐inducing medium containing 10% FBS, 10 mM β‐glycerophosphate (Sigma‐Aldrich), 0.1 μM dexamethasone (Sigma‐Aldrich), and 50 μg/mL ascorbic acid (Sigma‐Aldrich). The mineralization‐inducing medium was replaced every 2 days.

### 2.5 Real‐time PCR (RT‐PCR)

The TM Total RNA Kit I (OMEGA) was used to harvest total RNA from GMSCs following the manufacturer's instructions. Total RNA was reverse transcribed into cDNA using HiScript II Q RT SuperMix for qPCR (Vazyme). The cDNA amplification was performed using the ChamQ SYBR qPCR Master Mix (Vazyme) and the CFX96 Touch Real‐Time PCR Detection System (Bio‐Rad). The expression level of *Gapdh* was used as a standard to normalize the relative expression of each gene. The primers used for RT‐PCR are listed in Table 1.

**TABLE 1:** List of primers used for RT‐PCR.

Gene	Forward (5′‐3′)	Reverse (5′‐3′)
*Alp*	GTGACTACCACTCGGGTGAAC	CTCTGGTGGCATCTCGTTATC
*Bsp*	GACTTTTGAGTTAGCGGCACT	CCGCCAGCTCGTTTTCATC
*Ocn*	ATGGCTTGAAGACCGCCTAC	AGGGCAGAGAGAGAGGACAG
*Runx2*	GACTGTGGTTACCGTCATGGC	ACTTGGTTTTTCATAACAGCGGA
*Sox9*	CAGCCCCTTCAACCTTCCTC	TGATGGTCAGCGTAGTCGTATT
*Thy‐1*	CCAACCAGCCCTATATCAAGGT	TGAAGCTCACAAAAGTAGTCGC
*Vcam1*	TTGGGAGCCTCAACGGTACT	GCAATCGTTTTGTATTCAGGGGA
*Gapdh*	AGGTCGGTGTGAACGGATTTG	GGGGTCGTTGATGGCAACA

### 2.6 Western blotting

Total protein was harvested from GMSCs using radioimmunoprecipitation assay (RIPA) buffer containing protease inhibitors (Beyotime). Total protein concentration was determined using a BCA Protein Assay Kit (Beyotime). Sodium dodecyl sulphate–polyacrylamide gel electrophoresis (SDS–PAGE) sample loading buffer (Biosharp) was added to the protein samples from each group. The protein samples were denatured at 95°C for 10 min and separated using 10% SDS–PAGE gel. After separation, protein samples were transferred onto polyvinylidene difluoride (PVDF) membranes. Then, the PVDF membranes were blocked with blocking buffer for western blotting (Beyotime) and incubated with primary antibodies overnight at 4°C. Afterwards, the membranes were incubated with horseradish peroxidase (HRP)‐conjugated goat anti‐rabbit immunoglobulin G (IgG) (AS014, ABclonal, 1:10,000) for 1 h at room temperature. The Western Blot ECL HRP Substrate Kit (Advansta) was used to visualize the protein bands on the PVDF membranes following the manufacturer's recommendations. Quantitative analysis of all bands was performed using ImageJ version 1.53e (National Institutes of Health). GAPDH was used to normalize the relative expression levels of each protein. The primary antibodies included anti‐alkaline phosphatase (ALPL, A0514, ABclonal, 1:1000), anti‐β‐catenin (β‐catenin, A11932, ABclonal, 1:1000), anti‐bone sialoprotein (BSP, #5468, Cell Signaling Technology, 1:1000), anti‐runt‐related transcription factor 2 (RUNX2, A11753, ABclonal, 1:1000), anti‐SRY (sex‐determining region Y)‐box transcription factor 9 (SOX9, A2479, ABclonal, 1:1000), anti‐Thy‐1 (A2126, ABclonal, 1:1000), anti‐vascular cell adhesion molecule 1 (VCAM1, A0279, ABclonal, 1:1000) and glyceraldehyde‐phosphate dehydrogenase (GAPDH, PMK053M, Biopm, 1:10,000).

### 2.7 Alkaline phosphatase (ALP) staining

The GMSCs were inoculated into 12‐well plates. After 1 day of cell attachment, the medium was changed to a mineralization‐inducing medium. After 7 days of mineralization induction, 4% paraformaldehyde (Servicebio) was used to fix the cells. Each well was rinsed thrice with double‐distilled water. Finally, an ALP staining kit (Beyotime) was used to stain the cells, following the manufacturer's recommendations. The macro images were captured by a camera (D7200, Nikon) and the micrographs were taken by a microscope (CKX53, Olympus).

### 2.8 Alkaline phosphatase activity analysis

The GMSCs were seeded in 12‐well plates. When cell growth reached 80% confluence, the medium was changed to a mineralization‐inducing medium. After 7 days of mineralization induction, RIPA buffer containing a protease inhibitor was used to harvest the total protein. The BCA Protein Assay Kit (Beyotime) was used to measure the total protein concentration. The ALP activity of each group was determined using an ALP activity kit (Beyotime) following the manufacturer's recommendations. Then, ALP activity was quantified by measuring the absorption at 405 nm on a microplate reader (PowerWave XS2, BioTek). The data were standardized to the total protein concentration in each sample. Finally, relative ALP activity was normalized based on the average ALP activity of the blank group.

### 2.9 Alizarin red staining

The GMSCs were seeded in six‐well plates. The cells were then cultured in a mineralization‐inducing medium. After 21 days of mineralization induction, 4% paraformaldehyde was used to fix cells, which were then rinsed thrice with double‐distilled water. Afterward, the cells were stained with Alizarin red solution (Servicebio). The macro images were captured by a camera (D7200, Nikon), and the micrographs were taken by a microscope (CKX53, Olympus). Next, the alizarin red dye was solubilized using 10% hexadecylpyridinium chloride monohydrate (Servicebio) for 2 h. Finally, a microplate reader (PowerWave XS2, BioTek) was used to measure the absorption at 562 nm.

### 2.10 RNA sequencing

The TM Total RNA Kit I (OMEGA) was used to harvest total RNA from GMSCs, and its concentration and integrity were determined using the RNA Nano 6000 Assay Kit (Bioanalyzer 2100 system). Illumina sequencing and cDNA library construction were conducted by Beijing Annoroad Gene Technology Corporation. In brief, the generation of sequencing libraries was performed using the NEBNext_®_ Ultra**™** RNA Library Prep Kit for Illumina_®_ (#E7530L, NEB) according to the manufacturer's protocol. The libraries were sequenced on an Illumina platform with 150 bp paired‐end reads.

### 2.11 Cell counting kit‐8

The GMSCs were seeded in a 96‐well plate (5000 cells/well). After 12 h, various concentrations of PNU74654 (0, 10, 50, 100, 150 and 200 μM) were added into the medium. Afterward, Cell Counting Kit‐8 (CCK‐8) (Biosharp) was added into the medium in each well and incubated at 37°C for 2 h at the same time each day on days 0, 1, 3 and 5. A microplate reader (PowerWave XS2, BioTek) was used to determine absorption at 450 nm. Finally, the data was normalized as percentage of the control groups at each time point.

### 2.12 Statistical analysis

The quantitative results of the present study were presented as the mean ± standard deviation (SD). GraphPad Prism version 8.0.2 (GraphPad Software) was used to conduct statistical analyses. One‐way analysis of variance (anova) with Dunnett's test was used to compare the results for the two groups with those for the sh‐Thy‐1 group that was set as the control. *p < 0.033, **p < 0.002, ***p < 0.001 were considered statistically significant.

## 3 RESULTS

### 3.1 Characterization of GMSCs

GMSCs isolated from mouse gingiva were characterized by the presence of CD90 and CD105, markers of MSC, and the absence of CD19 and CD44 (Figure S1).

### 3.2 Effect of lentivirus on Thy‐1 knockdown of GMSCs

**FIGURE 1:**
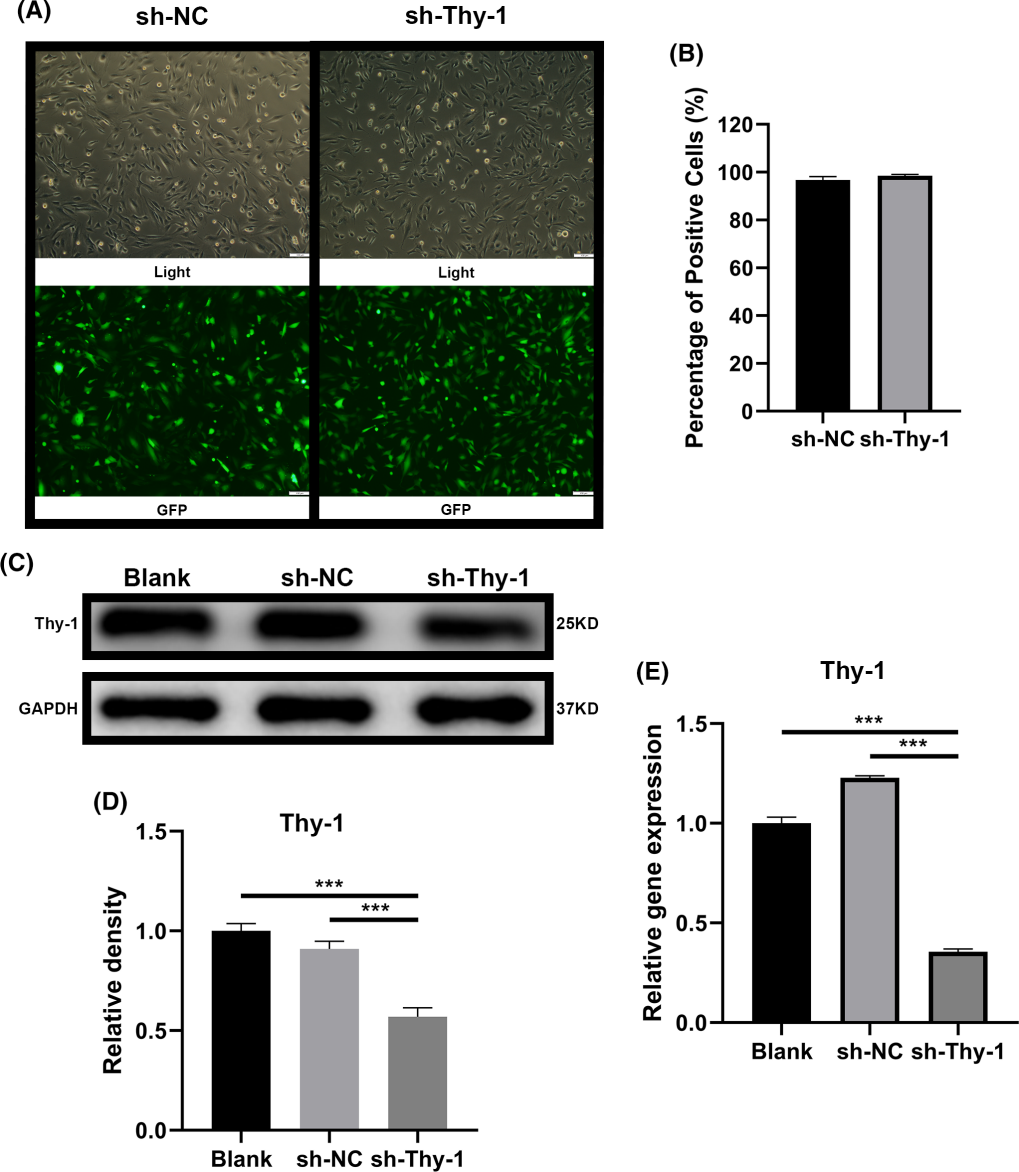
Expression of Thy‐1 in Thy‐1‐knockdown gingival mesenchymal stem cells (GMSCs). (A) Images of GMSCs transduced with sh‐Thy‐1 lentivirus or sh‐NC lentivirus were captured using a bright light (upper panels) or fluorescent (lower panels) microscope. (B) Percentage of positive GMSCs transduced with sh‐Thy‐1 lentivirus or sh‐NC lentivirus. (C, D) Representative western blot images and semi‐quantitative analysis of Thy‐1 protein expression. (E) Expression of *Thy‐1* gene detected using RT‐PCR. Data are expressed as means ± SD from at least three independent replicates. One‐way anova followed by a Dunnett test was used. Statistical significance is demonstrated as ***p < 0.001. Scale bar: 100 μm.

We reduced Thy‐1 expression in GMSCs by lentiviral transduction, which expressed shRNA Thy‐1. After transduction, GMSC lines that stably expressed shRNA Thy‐1 (sh‐Thy‐1) or shRNA (sh‐NC) controls were screened by antibiotic selection. Intracellular fluorescence intensity was used to determine shRNA expression in GMSCs. The results demonstrated a high intensity of GFP fluorescence in each cell of the sh‐Thy‐1 and sh‐NC groups (Figure 1A, B), indicating that GMSCs were successfully transfected with the lentivirus and expressed shRNA. RT‐PCR and western blotting confirmed that the expression of Thy‐1 in the sh‐Thy‐1 group was significantly reduced compared to that in the normal GMSC (blank group) and sh‐NC groups (Figure 1C, D, E).

### 3.3 Thy‐1 knockdown promoted the mineralization of GMSCs

**FIGURE 2:**
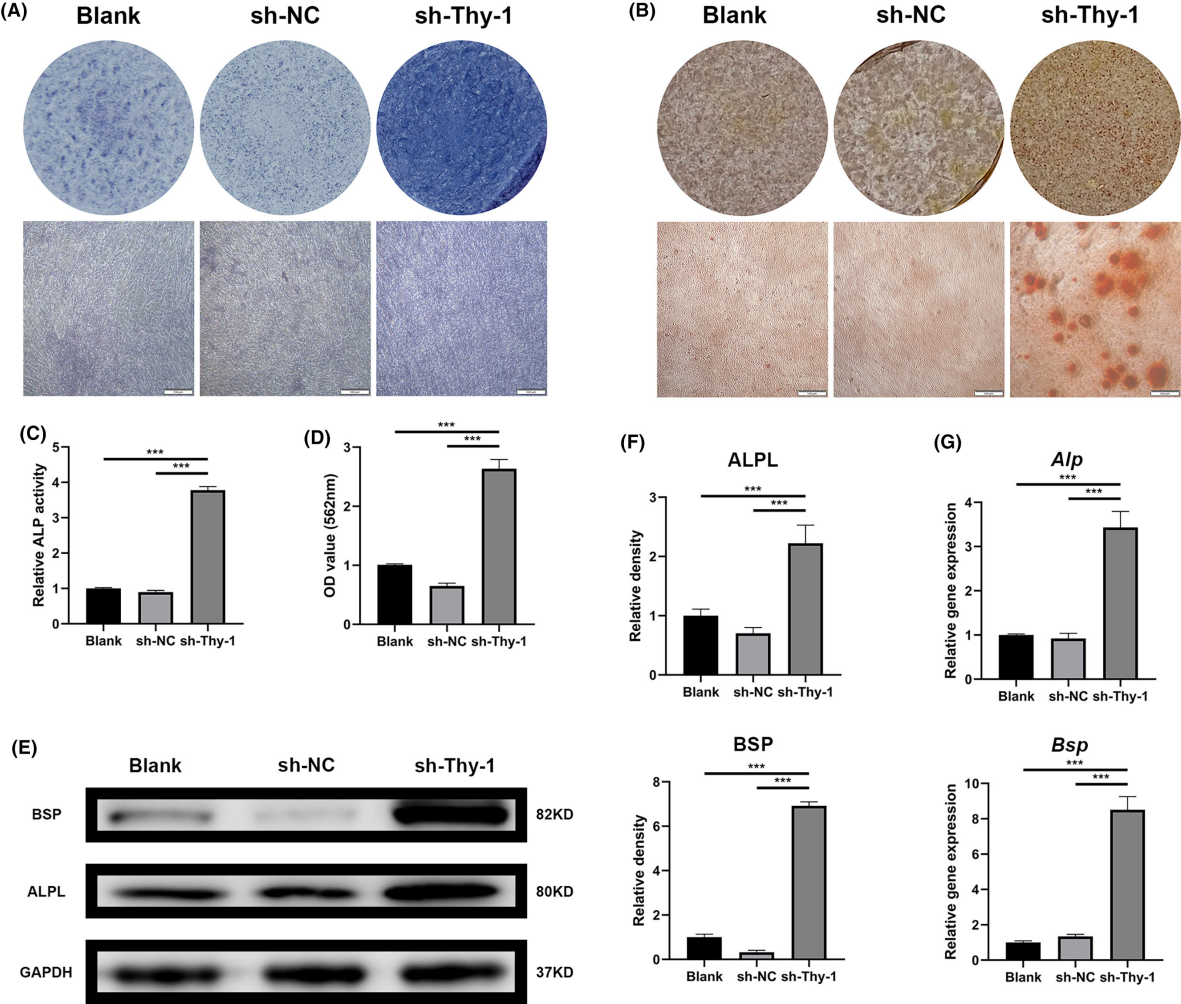
Effect of Thy‐1 on the mineralization of gingival mesenchymal stem cells (GMSCs). (A, C) Representative alkaline phosphatase (ALP) staining images and ALP activity analysis. (B, D) Representative Alizarin red staining images and semi‐quantitative analysis. (E, F) Representative western blots images and semi‐quantitative analysis of ALPL and BSP protein expression. (G) The expression of *Alp*, and *Bsp* genes detected using RT‐PCR. Data are expressed as means ± SD from at least three independent replicates. One‐way anova followed by a Dunnett test was used. Statistical significance is demonstrated as ***p < 0.001. Scale bar: 100 μm.

At the macrolevel, we observed that Thy‐1 knockdown led to a significant decline in ALP activity (Figure 2A, B) and the number of mineralized nodules (Figure 2C, D) in sh‐Thy‐1 cells. RT‐PCR and western blotting results demonstrated that the expression of ALP, BSP and the osteogenic markers in the sh‐Thy‐1 group was dramatically increased compared to that in the blank and sh‐NC groups (Figure 2E, F, G). Thus, Thy‐1 knockdown facilitated the mineralization of GMSCs.

### 3.4 Thy‐1 knockdown resulted in decreased expression of SOX9 and VCAM1

**FIGURE 3:**
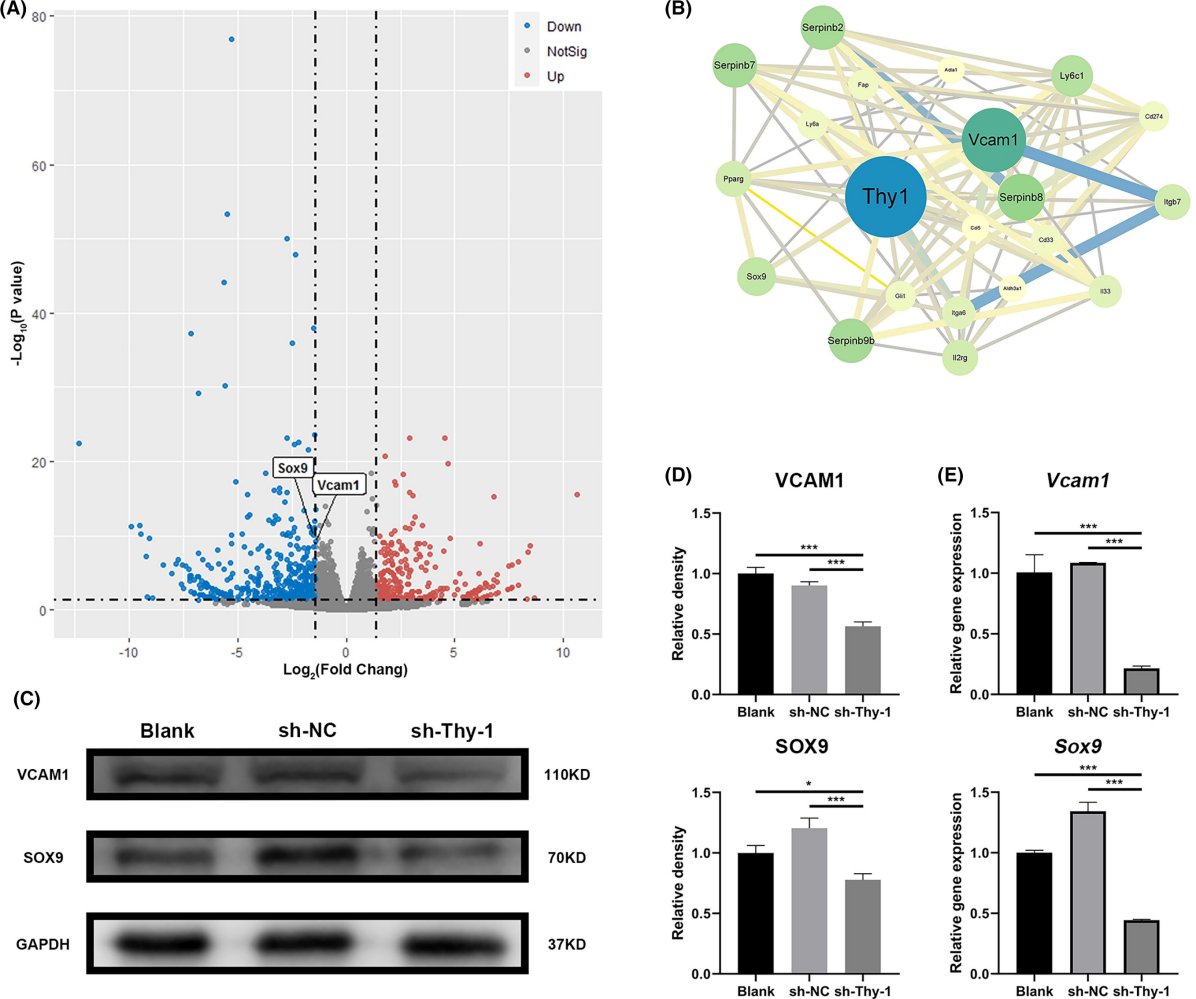
Thy‐1 knockdown reduced the expression of SOX9 and VCAM1. (A) RNA‐seq volcano map for the sh‐NC and sh‐Thy‐1 groups. (B) Protein interaction network analysis of genes with significant differences between sh‐NC and sh‐Thy‐1. (C, D) Representative western blot images and semi‐quantitative analysis of SOX9 and VCAM1 protein expression. (E) Expression of *Sox9* and *Vcam1* genes detected using RT‐PCR. Data are expressed as means ± SD from at least three independent replicates. One‐way anova followed by a Dunnett test was used. Statistical significance is demonstrated as *p < 0.033, ***p < 0.001.

To analyse the cause of the enhanced osteogenic function of GMSCs caused by Thy‐1 knockdown and identify possible signalling pathways, we used RNA‐seq methodology. Afterward, we performed an interaction network analysis of genes whose expression was notably different between the sh‐Thy‐1 and control groups. We screened for *Sox9* and *Vcam1*, which are associated with MSC osteogenesis (Figure 3A, B). RT‐PCR and western blotting results were further verified by the observation that the expression of SOX9 and VCAM1 was significantly lower in the sh‐Thy‐1 group than in the blank and sh‐NC groups (Figure 3C, D, E).

### 3.5 Thy‐1 knockdown promoted the mineralization of GMSCs via the Wnt/β‐catenin signalling pathway

**FIGURE 4:**
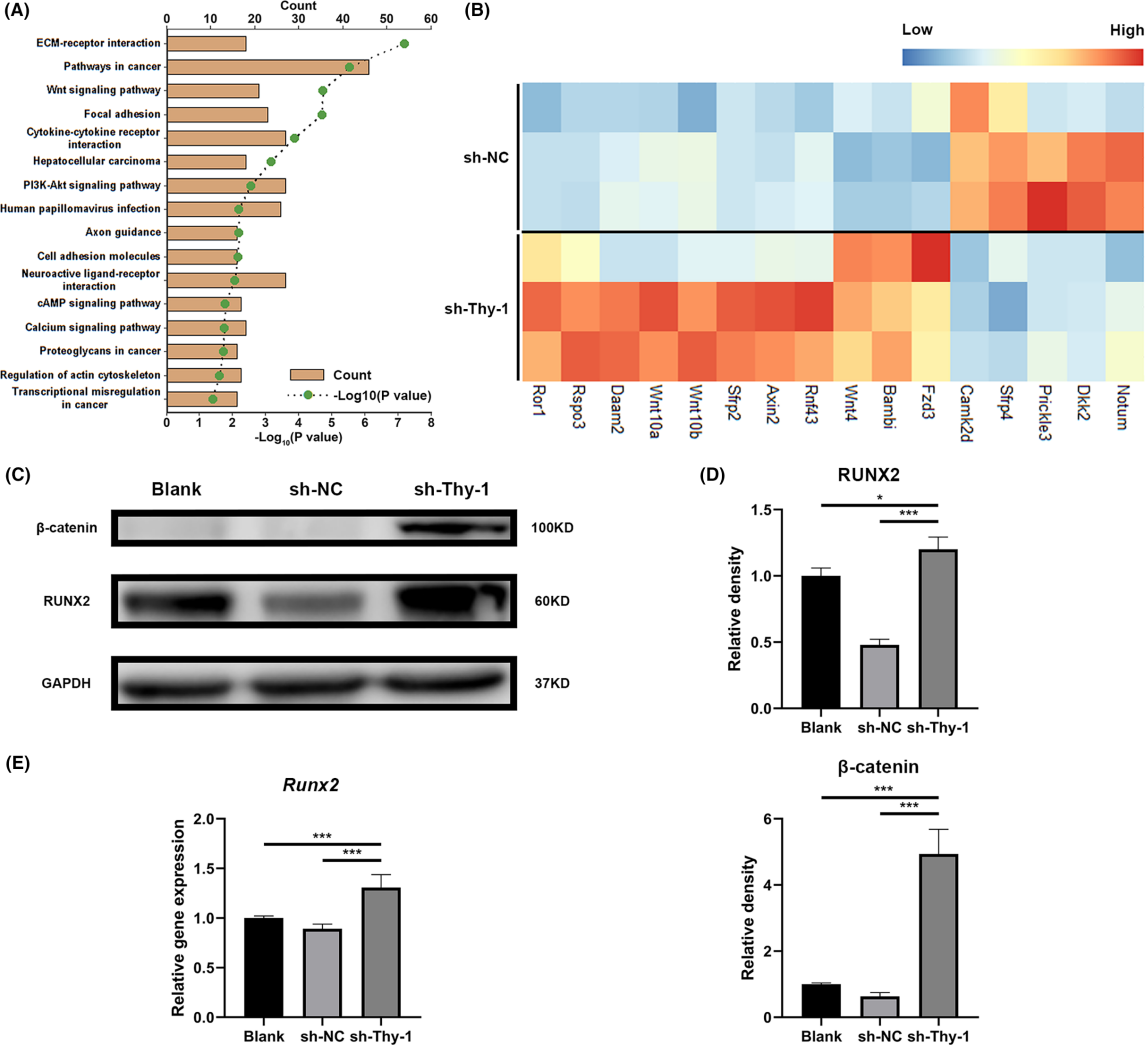
Effect of Thy‐1 on the signalling pathway of gingival mesenchymal stem cells (GMSCs). (A) KEGG analysis of genes with significant differences between sh‐NC and sh‐Thy‐1. (B) The expression of marker genes in the Wnt signalling pathway. (C, D) Representative western blot images and semi‐quantitative analysis of β‐catenin and RUNX2 protein expression. (E) Expression of *Runx2* gene detected using RT‐PCR. Data are expressed as means ± SD from at least three independent replicates. One‐way anova followed by a Dunnett's test was used for statistical analysis. Statistical significance is indicated as *p < 0.033, ***p < 0.001.

We further attempted to identify the signalling pathways involved in the increased mineralization of GMSCs following Thy‐1 knockdown. Kyoto Encyclopedia of Genes and Genomes pathway analysis indicated that some genes with significant differences between the two groups were enriched in the Wnt signalling pathway (Figure 4A, B). Further experiments confirmed that Thy‐1 knockdown resulted in increased expression of β‐catenin (Figure 4C, D), which contributed to the expression of RUNX2 (Figure 4C, D, E) and finally promoted the osteogenic ability of the sh‐Thy‐1 group.

**FIGURE 5:**
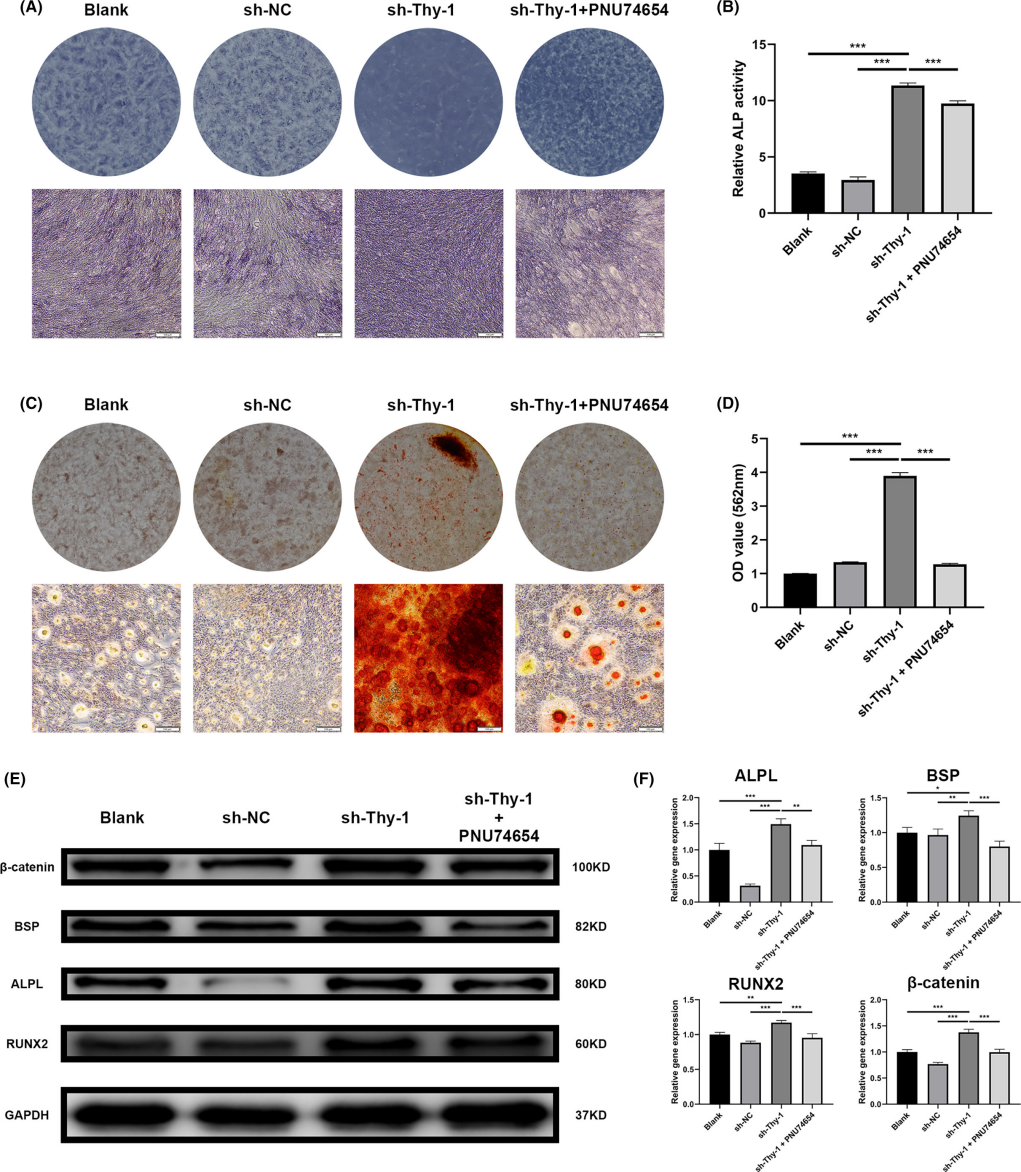
Thy‐1 regulated the mineralization of GMSCs via the Wnt/β‐catenin signalling pathway. (A, B) Representative alkaline phosphatase (ALP) staining images and ALP activity analysis. (C, D) Representative Alizarin red staining images and semi‐quantitative analysis. (E, F) Representative western blot images and semi‐quantitative analysis of ALPL, BSP, β‐catenin, and RUNX2 protein expression. Data are expressed as means ± SD from at least three independent replicates. One‐way anova followed by a Dunnett test was used. Statistical significance is demonstrated as *p < 0.033, **p < 0.002, ***p < 0.001. Scale bar: 100 μm.

We used PNU74654, a β‐catenin inhibitor, to inhibit the Wnt signalling pathway.^27^, ^28^ CCK‐8 assay results showed that GMSCs could still show some cellular activity in a medium containing 100 μM PNU74654 (Figure S2). To exclude the effect of proliferation, GMSCs from all groups were placed at a high density, and biomineralization was induced after 24 h. Treatment of the sh‐Thy‐1 group with PNU74654 abolished the osteogenesis‐promoting effect of Thy‐1 knockdown (Figure 5A, B, C, D). Western blotting results demonstrated that PNU74654 could reverse the increase in the expression of ALPL, BSP, RUNX2 and β‐catenin caused by Thy‐1 knockout (Figure 5E, F).

## 4 DISCUSSION

The use of bone tissue engineering to repair maxillofacial bone defects has become a frontier era of current research in regenerative medicine owing to the potential of restoring bone tissue from injury or damage to a normal and healthy state, among which stem cell‐based regeneration therapy has drawn broad attention.^29^, ^30^, ^31^ However, seed cell is one of the key problems restricting the development of bone tissue engineering.^32^ Our previous study indicated that GMSCs have the advantages of easy collection, rapid proliferation and a strong in vitro osteogenic differentiation potential.^7^ Multiple signalling molecules regulating the fate decision of MSCs have been reported; however, the complicated molecular mechanisms controlling the osteogenic differentiation of GMSCs remain incompletely understood.

In previous studies, Thy‐1 has been described as a key molecule mediating the fate decision of MSCs, playing a pivotal role between their adipogenic and osteogenic differentiation. In our previous study, Thy‐1 was presumed to be a possible cause for the differences in proliferative ability and osteogenic potential between BMSCs and GMSCs.^7^ However, the role of Thy‐1 in cellular differentiation remains controversial. Picke et al. showed that Thy‐1‐knockout mice were obese, with depressed bone formation and bone mass under high‐fat diet conditions.^33^ Further studies revealed that BMSCs from Thy‐1‐knockout mice showed enhanced adipogenic differentiation potential and reduced osteogenic differentiation potential compared to BMSCs from wild‐type mice.^25^ However, Varisco et al. argued the effect of Thy‐1 on adipogenic differentiation. The Thy‐1 transduction of into fibroblasts from Thy‐1‐deficient mice increased lipid accumulation, promoted the fatty‐acid uptake and facilitated the expression of peroxisome proliferator‐activated receptor γ (PPARγ).^34^ Moraes et al. used a lentivirus to transduce shRNA Thy‐1 into MSCs from different sources and found that the osteogenic capability of Thy‐1‐knockdown MSCs increased in ex vivo cultures.^26^ In the present study, we also used a lentivirus to knock down the expression of Thy‐1 in GMSCs and observed that Thy‐1 knockdown increased ALP activity and the number of mineralized nodules in GMSCs during mineralization induction. RT‐PCR and western blotting results showed that Thy‐1 knockdown enhanced the expression of mineralization markers. Thus, Thy‐1 knockdown significantly promotes the osteogenic differentiation of GMSCs, which is consistent with the findings of Moraes et al.^26^

To further investigate why Thy‐1 knockdown promotes the osteogenic potential of GMSCs, we used RNA‐seq to explore the effect of Thy‐1 on GMSC transcripts. These results demonstrated the declining expression of *Vcam1* and *Sox9*, which are considered to be related to the osteogenic differentiation of MSCs. VCAM1, also known as cluster of differentiation 106 (CD106), is a 90 kDa transmembrane glycoprotein^35^, ^36^ found in BMSCs, dendritic cells, activated vascular endothelium, bone marrow fibroblasts and tissue macrophages.^37^, ^38^ VCAM1 may play a vital role in regulating the osteogenic differentiation of MSCs; however, its regulatory mechanism remains unknown. *Vcam1* expression decreased with the osteogenic differentiation of BMSCs or osteoblasts.^37^, ^39^ The cross‐linking of VCAM1 and ICAM1 (intercellular adhesion molecule 1) on osteoblasts induces interleukin 6 (IL‐6) production, thereby inhibiting osteogenesis.^40^, ^41^ The osteogenic differentiation ability of VCAM1‐negative BMSCs is significantly higher than that of VCAM1‐positive BMSCs, suggesting that VCAM1 can be used as a marker to assess the differentiation ability of MSCs.^42^ Further, SOX9, a fatal transcription factor in chondrocytes, belongs to a family of 20 proteins called the SOX family.^43^, ^44^ It is also critically important for bone formation. Rutkovskiy et al. reported that SOX9 is necessary for the commitment of osteoblast precursors.^45^ However, high levels of SOX9 in hypertrophic chondrocytes result in impaired bone marrow formation and skeleton growth.^46^, ^47^ SOX9 inhibits the osteogenic differentiation of cells by reducing the expression of RUNX2 and binding to RUNX2 to suppress its function.^48^, ^49^ In this study, RNA‐seq results indicated that the decreased expression of *Vcam1* and *Sox9* caused by Thy‐1 knockdown may be the cause of the enhanced mineralization ability of GMSCs, which is negatively correlated with the osteogenic differentiation of MSCs.

The Wnt/β‐catenin signalling pathway mainly consists of Wnt proteins, members of a family of secreted molecules and β‐catenin, the key member of the pathway.^50^ This signalling pathway is crucial for regulating the fate decision of MSCs related to osteogenesis, which are involved in the stimulation of osteoblast genesis and function.^51^ Enhanced function of the Wnt/β‐catenin signalling pathway results in increased bone mass in mice.^52^ Cytological studies have shown that activation of this pathway promotes bone formation by increasing osteoblast emergence.^53^ The classical Wnt/β‐catenin signalling pathway can be blocked by SOX9 via interacting with β‐catenin to induce the degradation of β‐catenin.^54^, ^55^ Yang et al. used PNU74654, a β‐catenin inhibitor, to interrupt the Wnt signalling pathway, thus inhibiting osteogenesis and osteogenesis‐related marker expression in MSCs.^56^ In the present study, the RNA‐seq results revealed that the Wnt/β‐catenin signalling pathway was activated in the sh‐Thy‐1 group compared with that in the blank and sh‐NC groups. Further studies demonstrated that the β‐catenin protein expression level was high in the sh‐Thy‐1 group. PNU74654 reversed the promoting effect of Thy‐1 knockdown on the expression of osteogenic markers, implying that the enhancing effect of Thy‐1 knockdown on the osteogenic differentiation of GMSCs was achieved via the Wnt signalling pathway.

In conclusion, our study proved the enhancing effect of Thy‐1 knockdown on the osteogenic differentiation of GMSCs and revealed the impact of Thy‐1 knockdown on the Wnt/β‐catenin signalling pathway. Thy‐1 has a regulatory effect on the differentiation of GMSCs. Thy‐1 was confirmed to be the key molecule concatenating the three transcription factors associated with the trilineage differentiation of MSCs.^25^ Our study also emphasizes the potential ability of Thy‐1 cells to modify MSCs, facilitating the application of MSCs in tissue engineering.

## AUTHOR CONTRIBUTIONS

**Gufeng Liu:** Data curation (lead); formal analysis (equal); investigation (lead); methodology (supporting); visualization (equal); writing – original draft (equal). **Guixin Zhu:** Data curation (supporting); investigation (supporting); methodology (supporting). **Xiaoyi Wu:** Data curation (supporting); investigation (supporting). **Ziqiao Tang:** Investigation (supporting). **Wenjun Shao:** Investigation (supporting). **Min Wang:** Supervision (supporting). **Haibin Xia:** Supervision (lead). **QUAN SUN:** Conceptualization (equal); methodology (lead); validation (equal); writing – review and editing (equal). **Mingdong Yan:** Project administration (equal).

## FUNDING INFORMATION

This work was supported by the Fundamental Research Funds for Central Universities (Grant No. 2042022kf1173) and the National Natural Science Foundation of China (Grant No. 32370816).

## CONFLICT OF INTEREST STATEMENT

There is no conflict of interest, either directly or indirectly, in the article.

## Supporting information


Figure S1.

Figure S2.

## Data Availability

The data in this study are available from the corresponding author upon reasonable request.
