# Supplementary figures and images for: Thy‐1 knockdown promotes the osteogenic differentiation of GMSCs via the Wnt/β‐catenin pathway
Source: J Cell Mol Med. 2023 Oct 2;27(23):3805–15. doi: 10.1111/jcmm.17955 (PMC10718136; doi:10.1111/jcmm.17955)

**A**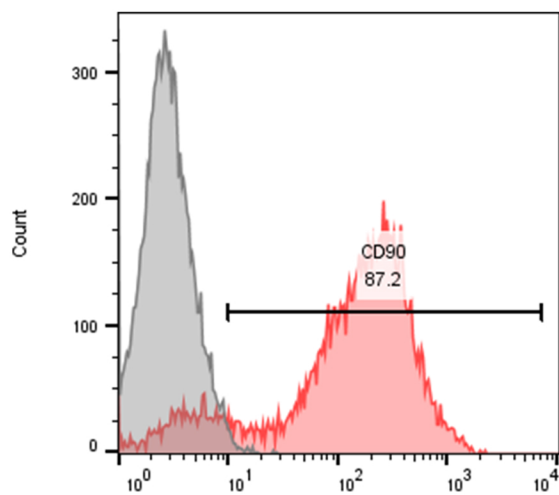**B**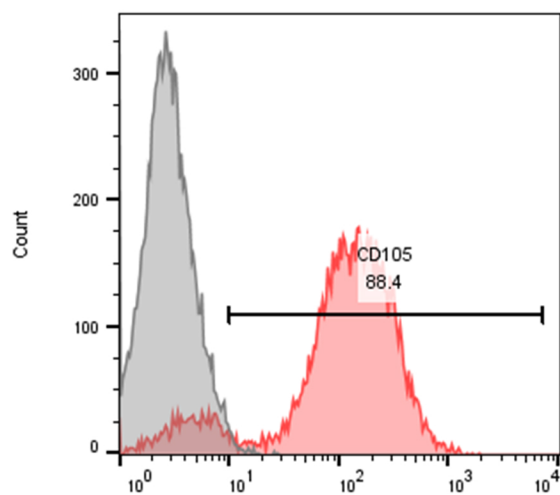**C**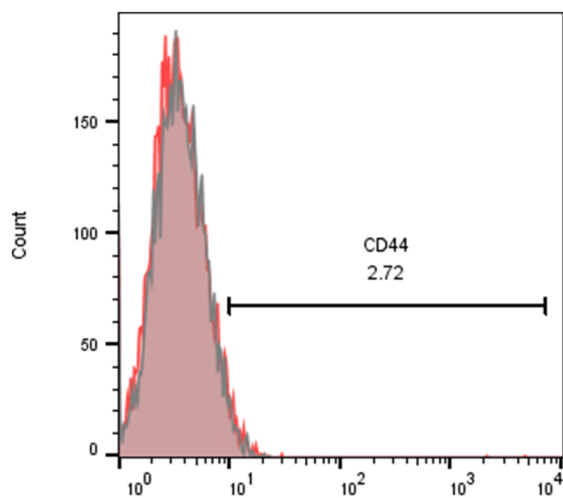**D**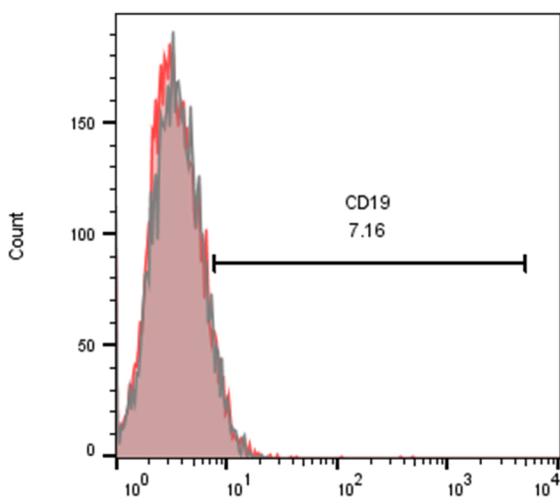

Supplement: Supplementary file 1 — Figure S1. [file JCMM-27-3805-s002.pdf]

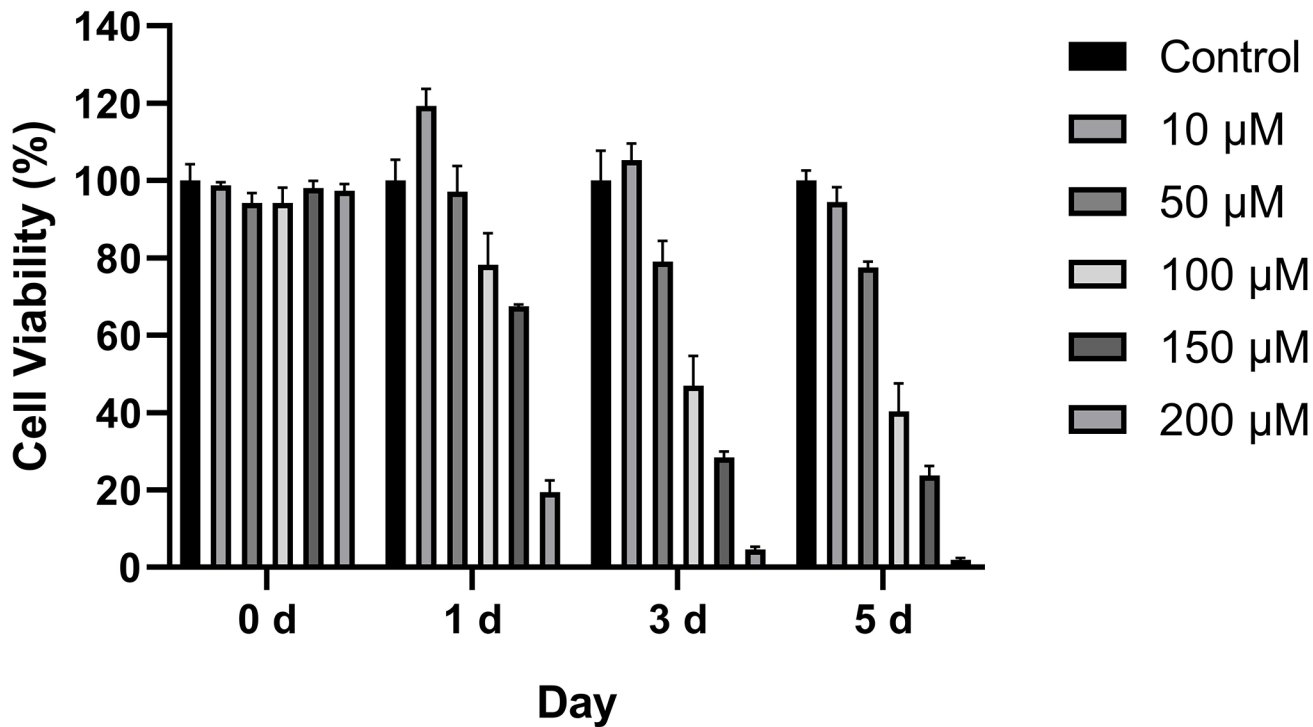

Supplement: Supplementary file 2 — Figure S2. [file JCMM-27-3805-s001.pdf]
